# Newly Licensed RNs Describe What They Like Best about Being a Nurse

**DOI:** 10.1155/2011/968191

**Published:** 2011-10-29

**Authors:** Maja Djukic, Linda H. Pellico, Christine T. Kovner, Carol S. Brewer

**Affiliations:** ^1^College of Nursing, New York University, 726 Broadway, 10th floor, New York, NY 10003, USA; ^2^School of Nursing, Yale University, 100 Church Street South, P.O. Box 9740, New Haven, CT 06536, USA; ^3^School of Nursing, University at Buffalo, 210 Wende Hall, 3435 Main Street, Buffalo, NY 14214-3079, USA

## Abstract

About 25% of newly licensed registered nurses (NLRNs) leave their first job within two years, but only 2% leave the nursing profession in this same timeframe. Therefore, the researchers sought to discover what new nurses like best about being a nurse, in hopes of gaining information that might help facilities to reduce turnover rates. Data were collected between January and March 2009 from 1,152 NLRNs licensed in 15 US states. Krippendorff's method was used to analyze survey responses. Five themes emerged: “providing holistic patient care,” “having an autonomous and collaborative practice,” “using diverse knowledge and skills to impact patient outcomes,” “receiving recognition,” and “having a job that is secure and stimulating.” Strategies are discussed that organizations might employ in helping NLRNs to realize what they best like about their work, which might lead to improved retention rates.

## 1. Introduction

Despite the favorable impact of the recent economic recession on the registered nurse (RN) shortage in hospitals, long-term solutions must be pursued in order to offset the estimated deficit of 260,000 RNs by 2025 [1]. Countering the shortage is essential; it has been well documented that adequate RN staffing has a positive impact on the safety and quality of care and on patient outcomes [2, 3]. Both recruitment and retention strategies have been employed to counter the shortage. Efforts at recruiting new nursing students have been so fruitful that about 54,000 qualified applicants from entry-level baccalaureate nursing programs were turned down in 2010 [4]. Conversely, retaining newly licensed RNs (NLRNs) in their jobs has not been as successful: US organizations fail to retain 26% of NLRNs for more than two years after the start of their first jobs, although almost 92% take another job in nursing [5]. This large discrepancy between organizational and professional turnover rates suggests that NLRNs are disenchanted with their employers but not with nursing. While the evidence on what professional and organizational characteristics are related to NLRNs' intentions of leaving their jobs is growing [6–8], we could not find any multistate studies in which NLRNs describe what they like best about being a nurse. Because improving NLRNs' retention rates decreases organizational costs [9] and ensures a stable future RN workforce [10], we hypothesize that creating opportunities for NLRNs to carry out what they best like about being a nurse might help to narrow the gap between professional and organizational turnover rates.

Those pursuing a nursing career believe that their work will provide them opportunities to help or care for others [11–14], as well as interesting, varied, and flexible employment prospects [13, 15, 16], good and stable income [14–16], and some degree of professional autonomy [15, 16]. Brennan [17, page 561] reported on the professional traits senior nursing students described as typical of nursing: “emotional proficiency” (e.g., being compassionate), “devotion/service” (e.g., being committed or an advocate), “dependability” (e.g., being part of a team), “independence” (e.g., being autonomous), “liability” (e.g., being accountable or a decision maker), and “cognitive proficiency” (e.g., being knowledgeable). Further, nursing education programs set expectations for future nurses to provide care that is patient centered, evidence based, high quality, and safe, delivered as part of an interdisciplinary team, and aided by information technologies [18, 19]. 

However, evidence suggests that many settings are not supportive of NLRNs providing the kind of care they expect to provide. For example, NLRNs have reported that excessive workloads can preclude the provision of patient-centered care [20–23]. They often describe work environments as unsafe [23] and their involvement in quality and safety initiatives as minimal [24]. Many NLRNs perceive colleagues to be antagonistic [20, 23, 25–27] and nurse managers as unsupportive [23, 28]. A study of a nationally representative sample of US RNs found that limited variety, autonomy, social support, collaboration with physicians, and opportunities for promotion and employment affected NLRNs' intentions of leaving their jobs [6].

Studies have described students' expectations of nursing practice prior to entering nursing school or starting a nursing job [11–17], the expectations nursing schools impose on them [18, 19], and their own discontent with practice environments [6, 20–28]. Lacking from the literature are NLRNs' insights into what the best part of nursing is that, if achieved, might help organizations to retain these nurses. This study aims to address this gap in knowledge. 

## 2. Design and Methods

In this descriptive study, we analyzed the responses to an open-ended question “What is the best part of being a nurse?” that we had inserted at the end of a quantitative survey designed to collect data on NLRNs' personal characteristics (such as education and marital status), work-environment and job characteristics (such as autonomy and job satisfaction), and employment opportunities. Information about the quantitative survey is provided elsewhere [28]. 

### 2.1. Sample

The institutional review boards at New York University and the University at Buffalo granted us permission to conduct the study. The random sample was NLRNs who answered the above question and were licensed by exam between August 1, 2007, and July 31, 2008, in metropolitan statistical areas and rural areas in 15 states (AL, KY, MD, MI, NC, NJ, NV, NY, OK, OR, PA, SC, TN, TX, and WV). The sample was designed to represent NLRNs residing in 25 metropolitan statistical areas (MSAs) and 2 rural counties in the 15 states. First, the distribution of NLRNs by each site needed in order to achieve the minimum desired sample size was determined. Each NLRN was assigned a random number within a site. This list was then organized by each of the 27 sites and sorted according to a random number. The first *N* number of respondents needed was selected from each site. For example, in the MSA site Greenville-Spartanburg-Anderson, 503 names and addresses of NLNRs were sent from the state. Individual randomly generated numbers were assigned to each of the 503 names. These were then sorted numerically (smallest to largest), and the first 190 names were selected. We recruited subjects from these 15 states because their nursing boards could identify RNs licensed by exam for the first time, and they provided data efficiently. Data were collected between January and March 2009. 

### 2.2. Data Analysis

Content analysis was used to identify the themes regarding what NLRNs reported as the best part of being a nurse. In content analysis, data are viewed as “representations not of physical events but of texts, images, and expressions that are created to be seen, read, interpreted, and acted upon for their meaning” [29, p. xiii]. Using Krippendorff's method, thematic units were chosen for this study because of their “descriptive richness” [29, p. 108]. Initially, an assistant entered into an Excel spreadsheet all of the NLRNs' written comments. The second author read all of the comments, so that a sense of the whole could be determined and coded the comments by selecting passages that related to the research question. Comments unrelated to the research question were not included in data analysis. The coded comments totaled nearly 41,000 words; individual comments ranged from two words (“job security”) to 324 words (average, 34 words). 

**Figure 1:**
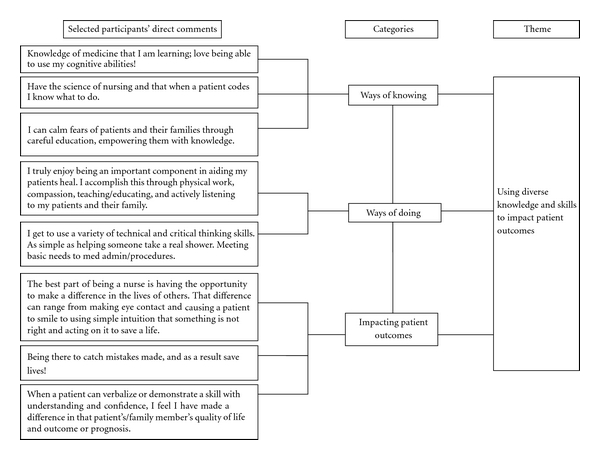
This is an example of a partial dendrogram showing how the respondents' comments were categorized and used in developing a theme.

The comments related to the research question were enumerated with the second author noting unique comments as well as recurrent passages. The participants' comments comprised of phrases and sentences were clustered or grouped to identify data that shared some characteristics and were then categorized. For example, phrases that came directly from participants such as “decreased pain” and “decreased nausea,” were characterized as “decreasing patients' symptomatology.” The participants' comments such as “the best part of being a nurse is being able to help people in time of need and saving lives every day” and “the feeling I get knowing I helped save a life or made a difference in a person's life is very gratifying for me” were characterized as “saving a patient's life.” Categories were developed from the selected comments, which required interpretation. For example, comments characterized as “decreasing patients' symptomatology” and “saving a patient's life,” were then categorized as “impacting patient outcomes.” Categories were clustered, and dendrograms (tree-like diagrams) were created to illustrate how participants' direct comments were collapsed into categories based on shared characteristics, and how the themes were derived from a cluster of categories. An example of a partial dendrogram is presented in Figure 1.

 Methodological integrity is important in considering the trustworthiness of findings. To that end, the second author created an audit trail to record reflections, evidence of consistency in coding, and interpretations of data. All of the authors reviewed the audit trail and had discussions about the selection of key characteristics, relationships, and the development of themes until an agreement was reached on the final coding scheme. In addition, numerous participant quotes were included in the results to enhance the credibility of the findings.

## 3. Results

Using the American Association of Public Opinion Research [30] response-rate definition (#3), the response rate for the quantitative survey was 57%, which resulted in a sample of 1,765. Of the 1,765 who responded to the quantitative survey, 1,195 answered the open-ended response question; of those, 43 comments were unrelated to the research question and included address or name changes. These comments were removed, resulting in an analytic sample of 1,152. As Table 1 shows, the NLRNs who provided comments had a mean age of 32.9 (SD = 9.1) years, with a range of 21 to 63 years. They were primarily white (75.6%), female (91.7%), employed full time (92.3%), and married (53.7%). Almost 85% were employed in inpatient hospital settings; 86.9% of the hospitals were not Magnet Recognition Program institutions. A majority of the respondents (59%) had an associate's degree at the time of entry into practice; the rest had a baccalaureate (37%), a diploma (3.4%), or a master's or doctoral degree (0.5%). Respondents reported a mean of 8.9 (SD = 4.2) months worked since licensure, with a range of 0 to 20 months. Although we sampled nurses from 15 US states in which they were first licensed, by the time of data collection, some subjects had already worked in another state. These additional 13 states were AZ, CA, CO, DE, FL, IN, KY, MN, MO, OH, VA, WA, and WI. 

Five themes emerged from the data describing the NLRNs' perceptions of what they considered to be the best parts of being a nurse: “providing holistic patient care,” “having an autonomous and collaborative practice,” “using diverse knowledge and skills to impact patient outcomes,” “receiving recognition,” and “having a job that is secure and stimulating.”

### 3.1. Theme One: Providing Holistic Patient Care

Overwhelmingly, NLRNs commented on the centrality of the nurse-patient dyad. Within this relationship, they said, nurses exercise “caring,” “compassion,” “kindness,” “helpfulness,” and “advocacy” for patients and families—aspects of their work that they described as paramount to their personal and professional contentment. One wrote that being a “care provider (both physical and mental aspect of care/need) and patient advocate and getting the patient trust of you is the most rewarding/important.” Another wrote, “Patient care is the best part of nursing.” 

The following comments suggest that these traditional nursing traits continue to give NLRNs a sense that they are “enriched,” “changed,” and “nourished.”

I believe that medical intervention is only a part of healing. It may edge on the side of “holistic” nursing but I try my best every time I'm at work to reflect a sense of caring, happiness, humor, confidence, and strength to my patients…. I have been put on this earth to do: nursing.

Feeling fulfilled in the process of helping others and believing I am attaining my purpose for this time in my life.

The best part of being a nurse is being able to help people in one of their times of great need. I am honored to be an advocate for my patients, to see that they are cared for in a way that restores them to health and is satisfactory to both the patient and their family members.

Many of the comments also suggest a spiritual component. Said one respondent “[nursing offers a] heightened awareness of the importance of spirituality & the reward from recognizing & embracing it.” Another noted, “Nursing is an outlet that allows me to serve and minister to others. I desire to give the best and most professional nursing care that I can give.” Many comments suggest that through providing holistic care, nurses grow personally.

### 3.2. Theme Two: Having an Autonomous and Collaborative Practice

In taking on the responsibility of ensuring that patients' needs are met, respondents said, NLRNs gain an awareness of their own autonomy.

I really enjoy the autonomy I have working as a nurse…. My input is very important and critical to the medical team.

I love the intensity and responsibility that comes with being a nurse.

Providing quality patient care, as a nurse in a Magnet^®^ hospital, it provides me with autonomy in my scope of practice.

Working in critical care I have a great deal of autonomy.

NLRNs liked the “nursing autonomy” they experienced. Yet they said that autonomy does not imply aloneness, but rather multidisciplinary collaboration with colleagues who value nurses' knowledge and contributions. Respondents noted that a collaborative environment “makes [the] job doable and more enjoyable; patients ultimately benefit.” The value of teamwork when faced with challenging working conditions is illustrated by the comment of an emergency room nurse.

At my job, the best part of being a nurse is the spirit of teamwork and collaboration between ER physicians and nurses and nurses with one another. We are constantly treating way too many patients at once, with limited supplies, inadequate space and stressful conditions. I rarely feel as if I don't have someone (coworkers) there to back me up. Also, because of doctors' trust in my assessment and treatment recommendation, I feel a valued part of the team.

### 3.3. Theme Three: Using Diverse Knowledge and Skills to Impact Patient Outcomes

The NLRNs' comments suggest that they gain satisfaction from using their knowledge and skills to improve patient outcomes. Their knowledge encompasses the “science of nursing,” the “nursing process,” “communication skills,” “a variety of technical & critical thinking skills,” “organizational skills,” and “know[ing] how,” as illustrated by the following comments.

The best part of being a nurse is having the opportunity to make a difference in the lives of others. That difference can range from making eye contact and causing a patient to smile to using simple intuition that something is not right and acting on it to save a life.

Knowing it's “my fault” a patient is pain free; helping patients smile in spite of the pain; watching patients' progress; the one-on-one contact with patients; helping patients of other languages/cultures to feel comfortable in a “foreign” setting; reading survey results that I did something right and made a difference for someone enough that they would remember days/weeks later.

The ability to educate and direct people toward healthcare and healthcare-related decisions. Having the knowledge to correctly answer questions about health-related matters or have the knowledge where to find answers I do not presently know.

In addition, respondents said that they believed that NLRNs are “providing quality care,” “using evidence-based practices,” and serving as “resources for both patients and colleagues.” The actions involved included “patient education,” “problem solving,” “critical thinking,” “making timely decisions that help patients,” and “catch[ing] mistakes.” 

They described the “honor,” “privilege,” and “joy” in witnessing the results of their care for patients, families, and the public. Participants commented on the outcomes of their care, including “patient coping,” “wellness,” “peaceful death,” “decreased pain,” “decreased nausea,” “finding cancer early,” “decreasing health care costs for the school district,” “contributing to improvement of community and public health,” “improved patients” understanding of their plan,” “ease [of] suffering,” “empowered patients,” and the “saving” of lives. The following quotes feature participants' recognition of the crucial role they play in the outcomes of delivering health care.

I feel a great sense of satisfaction and purpose from educating my patients on home care and providing support and a listening ear. When a patient can verbalize or demonstrate a skill with understanding and confidence, I feel I have made a difference in that patient's/family member's quality of life and outcome or prognosis.

Knowing that you may have changed a patient's life in a positive way. This can be as drastic as actually reviving a patient from a code, or as simple as being there when they needed to talk or make a life altering decision. RNs have a very powerful position in the care of a life. This should not be taken too lightly.

### 3.4. Theme Four: Receiving Recognition

The NLRNs' comments revealed the satisfaction they derive from their work and from the respect and gratitude they receive concerning their impact on patients, families, and communities. They wrote that the best part of being a nurse “is seeing satisfaction and gratitude on my patients' faces,” “the respect and admiration when people find out I am a nurse,” “professional respect,” “gratitude of patients and families,” and public “trust.” The following comments support this finding.

One memorable experience I recently had was a 24-week neonate admitted, and every care was given while the parents watched, sadly he did not survive the night but after handing mom her wrapped up child to hold, she stood up and gave me a big hug and said “Thank you for your hard work, watching your hard effort all night made this devastating experience a lot easier to deal with.” I started to tear and it was certainly a moment that strengthened me as a clinician/care giver and as a person.

The satisfaction of seeing my patient gets better—and knowing that your work was worth it and appreciated. It is amazing to watch the progression of critically ill patients; it honestly makes the long and stressful days worth every minute and every ache and pain!

### 3.5. Theme Five: Having a Job That Is Secure and Stimulating

The NLRNs' comments suggest that nursing provides professional and financial security. The NLRNs said that they appreciate the ability to find a position considered “recession proof” and flexible in terms of practice setting and schedule. Respondents expected “job security,” “competitive salaries,” and “professional advancement,” as illustrated by the following comments.

There are so many different areas provided for a registered nurse to pursue. Also with that aspect comes job security.

Before entering nursing, I worked in publishing. I left that field because I wanted to be able to positively affect others' lives, to work in a somewhat recession proof field, and be able to find a job no matter where I opted to settle. Luckily, I think all of those things still apply to nursing.

Job flexibility and career stability with current economic issues. The field is wide and one can change areas if burned in another area. Many opportunities for growth and diversifying careers.

Many of the comments addressed the “excitement” of the job, the changing patient populations, the continued learning, the “fast paced and unpredictable” nature of their work, and the challenges that “keep” them in the profession. They also said that nursing is “intellectually challenging” and that because “medicine is constantly changing” the “learning never stops,” as the following quotes demonstrate.

I enjoy the challenge—this is a job that requires me to use my education and critical thinking skills constantly. I also like that I pretty much see or learn something new every day.

I love… that it continually challenges me intellectually and emotionally. I love connecting with and supporting patients and families in crisis, amidst their vulnerability & tears. I never have to ask if my job matters. It is a joy to serve.

Having a profession that gives you a learning experience every time you are on the floor, you use your brain and common sense, you can make a difference to someone, there is a lot of room for growth and movement.

## 4. Discussion

The purpose of the study was to discover NLRNs' current views regarding the best part of being a nurse. The results are explicated in the following five themes.

**Theme One: Providing Holistic Patient Care**

It revealed that nurses value holistic and varied expressions of caring (physical, mental, psychosocial, spiritual), as well as the ability to advocate on patients' behalf. Similarly, Brennan [17, page 561] described caring and advocacy as traditional nursing attributes associated with “emotional proficiency” and “devotion/service.” Further, *ethos* is defined as the “disposition, character, or fundamental values peculiar to a specific person, people, culture, or movement” [31]. The nursing ethos encompasses caring, compassion, helpfulness, dignity, and empathy for the sick or well. In several studies, these traditional nursing characteristics were described as the ones that attract many students to nursing [11–14]. Our findings in Theme One suggest that NLRNs embody these traditional traits in the early stages of their practice.

**Theme Two: Having an Autonomous and Collaborative Practice**

It demonstrated NLRNs' appreciation of both autonomous and collaborative practice. The need for autonomy and teamwork aligns with Brennan's contemporary attributes of “independence” (such as being autonomous) and “liability” (such as being accountable or a decision maker) [17]. Others have reported that new nurses value autonomy and collegial relationships with physicians [6, 7, 15, 16, 23]. Further, such attributes continue to be incorporated into nursing education, as part of the quality and safety education for nurses (QSENs) framework and are influential in shaping nursing identity [18].

**Theme Three: Using Diverse Knowledge and Skills to Impact Patient Outcomes**

It showed that in their work NLRNs seek to apply a variety of skills, abilities, and types of knowledge, including, for example, communication, decision making, catching mistakes, critical thinking, problem solving, patient education, and intuition. The NLRNs reported the importance of being a source of evidence-based information for patients and colleagues making health care decisions. Providing care based on evidence fits the description by Brennan [17, p. 561] of role expectations such as “cognitive proficiency.” The diverse-role expectations that pertain to knowing and doing detailed in our study are also reported by Takase et al. [7, 8], who found that nursing-role expectations included the use of knowledge, decision making, and patient education.

We believe that a novel finding from our study pertains to the NLRNs' reported fulfillment from seeing how their nursing knowledge and skills contribute to improving outcomes for individuals and communities (for instance, decreasing health care costs for a school district). Unlike the attributes that the NLRNs describe as the best part of being a nurse in the other themes in our study which are already captured by available RN work environment instruments (e.g., [6]), the attribute from this theme is not. The NLRNs' focus on patient outcomes could be explained partly by recent efforts in nursing education to impart knowledge on the links between nurses' work and patient outcomes [18], as well as increased participation of nurses in data-reporting initiatives that link nursing care and outcomes, such as the National Database of Nursing Quality Indicators [32]. Future research should explore the relationship between staff retention and organizational capacity to engage staff RNs in continuous dialogue about the impact of nursing care on patient outcomes.

**Theme Four: Receiving Recognition and Theme Five, Having a Job That Is Secure and Stimulating**

It shows that new nurses find caring for patients is fulfilling, but they also need external rewards such as financial security, intellectual stimulation, and opportunity for professional advancement. Our findings echo those of other researchers who report that new entrants into nursing appreciate having job stability [14–16], and work that is flexible and stimulating [13, 15, 16].

### 4.1. Implications for Organizational Retention

We acknowledge that some turnover of NLNRs is unavoidable, but organizations can provide much of what the NLRNs said they liked about nursing, for example, investing in workflow redesign to allow NLRNs more time spent in direct patient care, a professional trait described in theme one that respondents said they value. Also, implementing some of the four high-performance workforce practices recommended by the Agency for Healthcare Research and Quality [33] could help meet certain needs that NLRNs identified in this study. For example, the use of teams in decentralized decision making [33] could help address NLRNs' need for autonomy and collaboration identified in theme two. McHugh et al. [33] also recommended tracking and rewarding performance related to patient outcomes, a strategy that might facilitate NRLNs' desire to see how their work affects patient care, as identified in theme three. Further, McHugh et al. [33] recommended the use of extensive training and skill development, as well as opportunities for career development. Suggestions include providing continuing education activities for employees and bringing in outside speakers if there is no resident expert, which aligns with the NLRNs' desire for professional growth, as identified in theme five. 

Organizations interested in retaining NLRNs could also consider implementing Transforming Care at the Bedside (TCAB), a model for engaging frontline staff in quality improvement initiatives. Bolton and Aronow [34] reported that TCAB initiatives in their facility resulted in a reduction in nurse-turnover rates from 7% to 3% over a four-year period, which was estimated to have saved the institution $5.6 million. The initiatives that were implemented included “physician–nurse rounding, physician–nurse education teams, employee recognition programs, letters of excellence awarded to top performers by the chief nursing officer, and interdisciplinary service agreements” [34, page 77-78]—all in harmony with desires the NLRNs identified in our study. 

### 4.2. Limitations

While the results contribute to the understanding of what NLRNs value in their work, the results should be generalized to the wider population of NLRNs with caution. This analysis is based on the comments of the NLRNs licensed in 15 US states who took the time to provide a written, qualitative response to a survey question. These NLRNs might have been particularly satisfied or dissatisfied with their jobs. Further, no independent coding of narrative responses was done by two authors. However, we think that we have used sound methods to ensure rigor in our data analysis. According to Sandelowski [35, page 33], auditability can be used as a “criterion of rigor or merit relating to the consistency of qualitative findings. A study and its findings are auditable when another researcher can clearly follow the “decision trail” used by the investigator in the study.” We believe that we have met this criterion for ensuring the rigor. In our case, the first, third, and the fourth authors all reviewed the second author's audit trail, and all of the authors had discussions about the selection of key attributes, relationships, and the development of themes until an agreement was reached on the final coding scheme.

## 5. Conclusions

Our findings, based on responses from NLRNs who have been in practice on average for less than nine month across 15 states, align closely with reported expectations of professional nursing practice for students planning to enter nursing education and anticipations for practice that education settings impart on their graduates. Despite the extraordinary advances in technology in recent years, the primacy of the nurse–patient dyad remains seminal to nurses' satisfaction. They recognize the impact of nurses in the lives of individual patients and in communities, and they are fulfilled by it. Additionally, they expect both financial and professional benefits for the provision of their services. The NLRNs' comments suggest that interprofessional collaboration is likely to not only result in improved patient outcomes but also in better nurse retention. Further, NLRNs recognize the need for continued development of clinical competencies and of formal education, and they expect their employers' support of this need. This is especially important given that “schools cannot adequately prepare students for practice in all places; each practice site demands that nurses learn a significant amount of local, specific knowledge” [36, page 31]. Benner et al. [36] suggest that this additional preparation for nursing practice could be accomplished as part of one-year residency programs in specialty work settings, coupled with improvements in undergraduate nursing education. The effectiveness of these interventions for improving NLRNs' retention should be studied in the future.

Organizations might be able to improve the retention of NLRNs within the first two years of starting their jobs by securing opportunities for new nurses to realize what our respondents described as the best parts of being a nurse: providing holistic care, being accountable and collaborative with other providers, seeing how their care is affecting patient outcomes, being recognized and rewarded for their work, and stimulated in their clinical environment. Creating opportunities for NLRNs to do what is most enjoyable in everyday practice may close the gap between the disproportionately large organizational versus professional turnover of NLRNs. Future research should examine the impact of organizational strategies on retention rates of NLRNs in a variety of settings.

## Figures and Tables

**Table 1:** Sample demographics (*N* = 1152).

Characteristic	*M *(SD)	%
Age (years)	32.9 (9.1)	—
Total months worked since passing NCLEX	8.9 (4.2)	—
Female		91.7
White		75.6
Black		11.1
Asian		5.4
Other		6.6
Married		53.7
Diploma		3.4
Associate degree		59.0
Baccalaureate		37.0
Masters/doctoral		0.5
Full time		92.3
Hospital (inpatient)		84.5
Hospital (outpatient)		4.0
Other		11.6
Employed in a Magnet^®^ accredited hospital		13.1
